# Bullet-related bacterial wound infections among injured personnel at emergency site hospitals in Bahir Dar: prevalence, antimicrobial susceptibility and associated factors

**DOI:** 10.1186/s12866-024-03324-2

**Published:** 2024-05-16

**Authors:** Enanu Tigabu, Addisu Melese, Feleke Mekonen, Yesuf Siraj

**Affiliations:** 1 Department of Medical Laboratory Sciences, GAMBY Medical and Business College, Bahir Dar, Ethiopia; 2 Department of Medical Laboratory Sciences, School of Health Sciences, College of Medicine and Health Sciences, Bahir Dar University, Bahir Dar, Ethiopia

**Keywords:** Bullet-related wound infection, Antimicrobial susceptibility profile, Risk factors

## Abstract

**Background:**

Bullet-related bacterial wound infection can be caused by high-velocity bullets and shrapnel injuries. In Ethiopia, significant injuries were reported that may cause severe wound infections, persistent systemic infections and may lead to amputation and mortality. The magnitude, antimicrobial susceptibility profiles, and factors associated with bacterial wound infections among patients with bullet-related injuries are not yet studied particularly at health facilities in Bahir Dar, Northwest Ethiopia. Therefore, this study was aimed to determine the prevalence, bacterial profiles, antimicrobial susceptibility profiles, and factors associated with bacterial infections among patients with bullet-related injuries at referral health facilities in Bahir Dar, Northwest Ethiopia.

**Methods:**

A Hospital-based cross-sectional study was conducted among patients with bullet-related injuries at three referral health facilities in Bahir Dar from May 25 to July 27, 2022. A total of 384 patients with bullet-related injuries were included in the study. Sociodemographic and clinical data were collected using a structured questionnaire. Wound swabs were collected aseptically and cultured on Blood and MacConkey agar following bacteriological standards. Biochemical tests were performed to differentiate bacteria for positive cultivation and antimicrobial susceptibility profiles of the isolates were done on Muller Hinton agar using the Kirby-Bauer disk diffusion technique according to the 2021 Clinical Laboratory Standard Institute (CLSI) guideline. The data were entered using Epi-Info version 7.3 and analyzed using SPSS version 25. Descriptive data were presented using frequency, percentages, figures, and charts. Logistic regression was carried out to identify factors associated with bacterial wound infections. *P*-value < 0.05 was considered statistically significant.

**Results:**

The prevalence of bullet-related bacterial wound infection among three referral hospitals in Bahir Dar city was 54.7%. The most commonly isolated Gram-negative organism was *Klebsiella spps* 49 (23.3%) while among Gram-positive organism, *Staphylococcus aureus* 58 (27.6%) and coagulase-negative staphylococci (CONS) 18 (8.6%). Contamination, hospitalization and smoking habit were significantly associated with the presence of bullet-related bacterial wound infections. Over 97% multidrug resistant (MDR) bacterial isolates were identified and of theses, *E. coli*, *Proteus* species, *Citrobactor*, and *Staphylococcus aureus* were highly drug resistant.

**Conclusion:**

Increased prevalence of bullet-related bacterial wound infection was noticed in this study. *S. aureus* followed by *Klebsiella* species were most commonly isolated bacteria. High frequency of resistance to Ampicillin, Oxacillin, Cefepime, Ceftriaxone, Ceftazidime, Vancomycin, and Norfloxacin was observed. Therefore, proper handling of bullet injuries, prompt investigation of bacterial infections, monitoring of drug sensitivity patterns and antibiotic usage are critical.

## Background

Physical damage to the skin is one of the major establishement of wound [1] where microbial pathogens can get access to deep skin tissues and cause wound infections [2, 3]. Bullet-related wound infection is occurred as a result of high speed bullet skin damage coupled with microbial contamination raised from military trenches [4].

When a fired bullet hits a person, the jacket of the bullet may remain in the cutaneous tissue and may continues to pass through subcutaneous, muscle and bone to produce severe wound. The wound then widens the breaks of the continuity of the skin and gives opportunity to several pathogens to multiply and cause infection [5]. Multiplication of pathogens in the injured tissues results a local wound infection that may lead to serious bullet-related health issues than from wounds inflicted by bullets [6, 7]. During injury, the shrapnel damages tearing flesh, breaking bones, and usually causes pointed irregular wounds and causes unavoidable sepsis [8], and becomes a serious global cause of morbidity and mortality [9].

Antibiotics are commonly used immediately upon arrival to the health institution to prevent the occurence of bullet-related wound infection [10]. However, some studies showed that bacteria can contaminate the wound immediately after injury [11] and obviously during hospitalization [12]. Since bullet-related wounds are different from other traumatic injuries due to the higher velocity of projectiles or blast devices, more severe injuries and wounds are often contaminated by clothing, soil, and environmental debris [10, 13]. Although several advancements have been made to abate combat-related mortality and case fatality rates (CFR), complications remain the main cause of morbidity and mortality of combat-injured personnel [14].

Furthermore, continual evolution of battlefield tactics that lead to new mechanisms of injury managenet and echo pattern trends to prevent complications of wound infections [15]. However, severe wound infections and antimicrobial resistance continue to rise and apparently need sustainable solutions [16, 17]. In developing countries like Ethiopia where trauma centers are not sufficiently available during war time, significant bullet-related injuries have been observed where persistent wound or systemic infections may lead to extremity amputation [18]. In bullet-related injuries, rapid surgical interventions can be primarily carried out using an amputation procedure to prevent life-threatening infections followed by microscopic identification of microorganisms and characterization of wound flora [2, 19] to initiate antimicrobial treatment.

Eventhough antimicrobial therapy is often used for perioperative prophylaxis and treatment of wound infections, a global rate of antimicrobial resistance is ever growing serious threat [5, 19] which also worsens the health conditions of bullet injured patients. This is the most challenging problem in fragile and conflict-affected regions [20]. Therefore, the current study was designed to assess the prevalence, antimicrobial susceptibility profiles, and factors associated with bacterial wound infections among patients with bullet-related injuries at three emergency site hospitals in Bahir Dar city, Northwest Ethiopia.

## Methods

### Study design, period, and area

A hospital-based cross-sectional study was conducted from May 25 to July 27, 2022 at Felege Hiwot Comprehensive Specialized Hospital (FHCSH), Tibebe Ghion Specialized Hospital (TGSH) and Northwestern Military Command Level 3 Hospital (NMCL3H), Bahir Dar, Northwest Ethiopia. During conflict seasons, FHCSH, TGSH and NMCL3H served about 2000, 3000 and 6000 injuries per month in their surgical wards, orthopedic wards, and others including emergency tents.

### Study participants and eligibility criteria

All patients with bullet-related injury attending at Bahir Dar city hospitals were the source population. The study populations were bullet-injured patients who were clinically diagnosed in the orthopedic and surgical wards of these hospitals during the study period. All bullet-related injured patients at selected hospitals in Bahir Dar city were included in the study. Unconscious patients and injuries not related to the bullet injury were excluded from the study.

### Sample size determination and sampling technique

The sample size was calculated by EPI Info 7.3 for a single population proportion based on the following assumption. Since bullet-related wound infections in Ethiopia was not studied, we considered its prevalence could be an 50% with a 95% confidence level and a margin of error of 5%. Using a single population proportion formula, a total of 384 bullet-related injured patients were included in the study.

### Study variables

Wound infection was a dependent variable while socio-demographic factors, personal habits, hospitalization stay, possible contamination history during injury, previous antibiotics use for prophylaxis and before the operational procedure, anatomical location of the wound, bacterial isolates and their antimicrobial susceptibility profiles and types of bullets for injuries were independent variables.

### Data collection

A questionnaire was developed after consulting previous publications and customized for data collection. Socio-demographic and clinical data were collected using face-to-face interviews and medical charts. Data were collected from each casualty including types of bullet of injury, location of injury, and antibiotics used.

### Specimen collection, transportation, and processing

Swab samples from wound pus were aseptically collected from the injured site of wound infection of bullet-related injured patients using sterile swabs before cleaning the wound. Each collected wound swab was innoculated into a tube containing Stuart’s transport medium (Oxoid, UK) and transported to the Microbiology Laboratory, Department of Medical Laboratory Sciences, College of Medicine and Health Sciences, Bahir Dar University using a cold-chain vaccine carrier.

### Bacterial culture techniques

The specimens were plated onto blood agar (Oxoid UK) and MacConkey (Oxoid UK) agar plates using a sterile wooden swab and wire loop. Innoculated agar plates were incubated at 37 °C in ambient air. The plates were used to grow gram-positive cocci and gram-negative rods. Growth was inspected at 24 h.

### Bacterial identification

Gram staining was performed from the grown pure colony and based on the gram reaction, the identification of Gram-positive bacteria were further characterized by catalase and coagulase tests. Gram-negative rods were identified based on their gram reaction and colony characteristics. Once isolates of Gram-negative bacteria (GNB) were isolated, they were subjected to various biochemical tests for species identification. Enterobacteriaceae were identified by H2S (Hydrogen Sulfide) and gas production in TSI (Triple Sugar Iron) agar, citrate utilization, urease test, sulfur indole motility test, oxidase, and carbohydrate utilization tests following standard operational procedures (SOPs).

### Antimicrobial susceptibility testing

The antibiotic sensitivity test for the isolated organism was determined by Kirby- Bauer disc diffusion method. Bacterial inoculums were prepared from 3 to 5 pure colonies by suspending the freshly grown bacteria in 5 ml sterile saline. The suspension was compared with turbidity equivalent to 0.5 McFarland standards and was streaked on the entire Muller-Hinton agar (Oxoid, UK) then, antimicrobial-impregnated paper disks were placed on the plate and incubated aerobically at 37˚C for 24 h and the results were interpreted according to CLSI (2021) guideline [21]. The zone of inhibition was measured by calibrated ruler and interpreted as sensitive, intermediate or resistant by using a standard chart. Antibiotics were selected based on accordance with availability in the market, frequent prescription and based on CLSI, 2021 guidelines. These antibiotics were Cefepime (30 µg), Ampicillin (10 µg), Ceftriaxone (30 µg), Gentamicin (10 µg), Norfloxacin (10 µg), Ceftazidime (30 µg), Ciprofloxacin (5µ), Vancomycin (10 µg), Oxacillin (30 µg), Tetracycline (15 mg) and Erythromycin (30 µg). Vancomycin and Oxacillin were used only for Gram-positive cocci isolates.

### Data quality control

The reliability of the study findings were guaranteed by implementing quality control (QC) measures throughout the whole process of the laboratory procedures. All materials, equipment, and procedures were adequately controlled and each procedure was aseptically performed. In addition, culture media were tested for sterility and performance. The sterility of the media was checked by incubating 5% of prepared media at 37˚C for 24–48 h. If there were no growth of bacteria on the prepared media, the procedures were continued. If there were the growth of bacteria, the whole batch of media were discarded. Growth performance of the media was checked by inoculating control strains of American Type Cell Collection (ATCC) *E. coli* 25922, *P. aeruginosa* ATCC 27853, and *S.aureus* ATCC 25923. The control strains were kindly provided from Amhara Public Health Institute (APHI). To standardize the inoculum density of bacterial suspension for the susceptibility test, a barium sulfate (BaSO4) turbidity standard, equivalent to a 0.5 McFarland standard was used.

### Data analysis

Data were cleaned, coded, and entered into EPI info 7.3 and exported to SPSS statistical software version 25 for analysis. Descriptive statistics such as socio-demographic, and clinical characteristics of the study participants along with bacterial isolates and their antimicrobial resistance (AMR) profile were calculated. Logistic regression analyses were used to test the relationship between the dependent variable and independent variables. Variables that showed a p-value < 0.25 in univariate logistic regression were selected for further analyses using multivariable logistic regression to avoid the effect of confounding factors. The frequency and percentages of bacterial isolates were calculated and p-value < 0.05 at a 95% confidence interval were considered statistically significant. Finally, the results were presented in words, graphs, and tables.

### Ethical approval

#### Ethical approval

was obtained from the ethical review committee of the College of Medicine and Health Science, Bahir Dar University (protocol No. 387/2022) prior to the commencement of the study. Permission letters obtained from APHI were given to the selected hospitals where the study population were admitted. Study participants were asked for verbal consent and written consent forms were obtained for full enrolnment. Study participants with positive culture results and AST were communicated with their caregivers (physicians, health officers or nurses) at the hospital for appropriate treatment and possible medical care. Subject confidentiality and any other special data security requirements were maintained and assured. Subject confidentiality was maintained by getting informed concent, avoiding personal information, identities and adresses and any other special data security requirements were assured.

## Results

### Demographic and clinical profile of patients with bullet-related bacterial wound infections

A total of 384 specimens were collected from bullet-related injured patients from May 25 to July 27, 2022. In this study, the prevalence of bullet-related bacterial wound infections in the study population was 54.7%. Among 384 bullet-injured patients included in the study, 199 (51.8%) were in the age range of 14–24 years with a mean ± SD age of 31.7 ± 1.14 and 368 (95.8%) were male. Of the total of 384 injured patients, 318 (82.8%) were Amhara in ethnicity, 321 (83.5%) were atleast able to write and read, 206 (46.4%) were study subjects who never drunk alcohol and most of them were never smoking cigarettes (319(83.1%)) and chewing khat (336(87.5%)) (Table 1).

**Table 1:** Socio-demographic characteristics of patients with a bullet-related bacterial infection in Bahir Dar, Northwest Ethiopia (*N* = 384)

Variables		Frequency	Percent
Age	14–24	199	51.8
	25–34	94	24.5
	35–44	50	13.0
	45–54	28	7.3
	55–64	11	2.9
	65–74	1	0.3
	75–84	1	0.3
Sex	Male	368	95.8
	Female	16	4.2
Health Facility	FHCSH	205	53.4
	TGSH	113	29.4
	NWCL3BH	66	17.2
Ethnicity	Amhara	318	82.8
	Oromo	34	8.9
	Other	32	8.3
Marital status	Single	187	48.7
	Married	192	50.0
	Divorced	1	0.3
	Widowed	4	1.0
Educational status	Not write and read	63	16.4
	Write and read	111	28.9
	Grade 1–6	103	26.8
	Grade 7–12	100	26.0
	Above grade 12	7	1.8
Alcohol drinking	Yes	178	46.4
	No	206	53.6
Cigarette Smoking	Yes	65	16.9
	No	319	83.1
Khat Chewing	Yes	48	12.5
	No	336	87.5

In this study, of the total of 384 injured patients, 251 (65.2%) were contaminated with soil during injury, and the majority of patients were injured their hands and legs (147 (38.3%)) followed by the abdomen (83 (21.6%)), and head and neck (70 (18.2%)). The mechanism of bullet-related wound injury were mostly occurred by Kalashnikov-bullet (149 (41.8%) and sniper bullet (51 (13.3%)) followed by other bullets. During this study, 103 (28.8%) bullet-injured patients were admitted for 60 days in the hospital while 53 (13.8%) were for 90 to 180 days. Of the total 384 injured patients, 234 (60.9%) started antibiotics delayed after injury (Table 2).

**Table 2:** Clinical and microbiological characteristics of patients with bullet-related bacterial wound infections (*N* = 384)

Variable	Frequency	Percent
Contamination		
Soil	251	65.4
Water	24	6.3
Dust	62	16.1
Mud	18	4.7
Other	29	7.6
Wound location		
Chest and back	70	18.2
Abdomen	83	21.6
Head and neck	62	16.1
Extremity (upper or lower)	21	5.5
Hand & leg	147	38.3
More than 2 locations	1	0.3
Types of bullet		
Blast	35	9.1
Gunshot (Pistil)	50	13.0
Artillery bullet	6	1.6
Sniper bullet	51	13.3
SKS –bullet	9	2.3
Kalashnikov-bullet	159	41.4
Mortar bullet	15	3.9
Rocket bullet	2	0.5
Other	57	14.8
Hospitalization Stay		
7 days	73	19.0
15 days	80	20.8
30 days	75	19.5
60 days	103	26.8
90–180 days	53	13.8
Previous use of antibiotics		
As soon as injured	150	39.1
Delayed After injury	234	60.9

Among the total of 210 positive cultures of swab specimens collected from three selected hospitals in Bahir Dar city, 120 (58.5%) were isolated from bullet-injured patients in FHCSH, 56 (49.5%) were in TGSH and 34 (51.5%) were in NMCL3H (Table 3).

**Table 3:** Culture positivity of bullet-related wounds of study subjects at selected three hospitals, in Bahir Dar Northwest Ethiopia (*N* = 210)

Hospital	Culture positive	
	Number	Percent
FHCSH	120	58.5
TGSH	56	49.5
NWCL3BH	34	51.5

### Bacterial profile of bullet-related wound infections

**Fig. 1:**
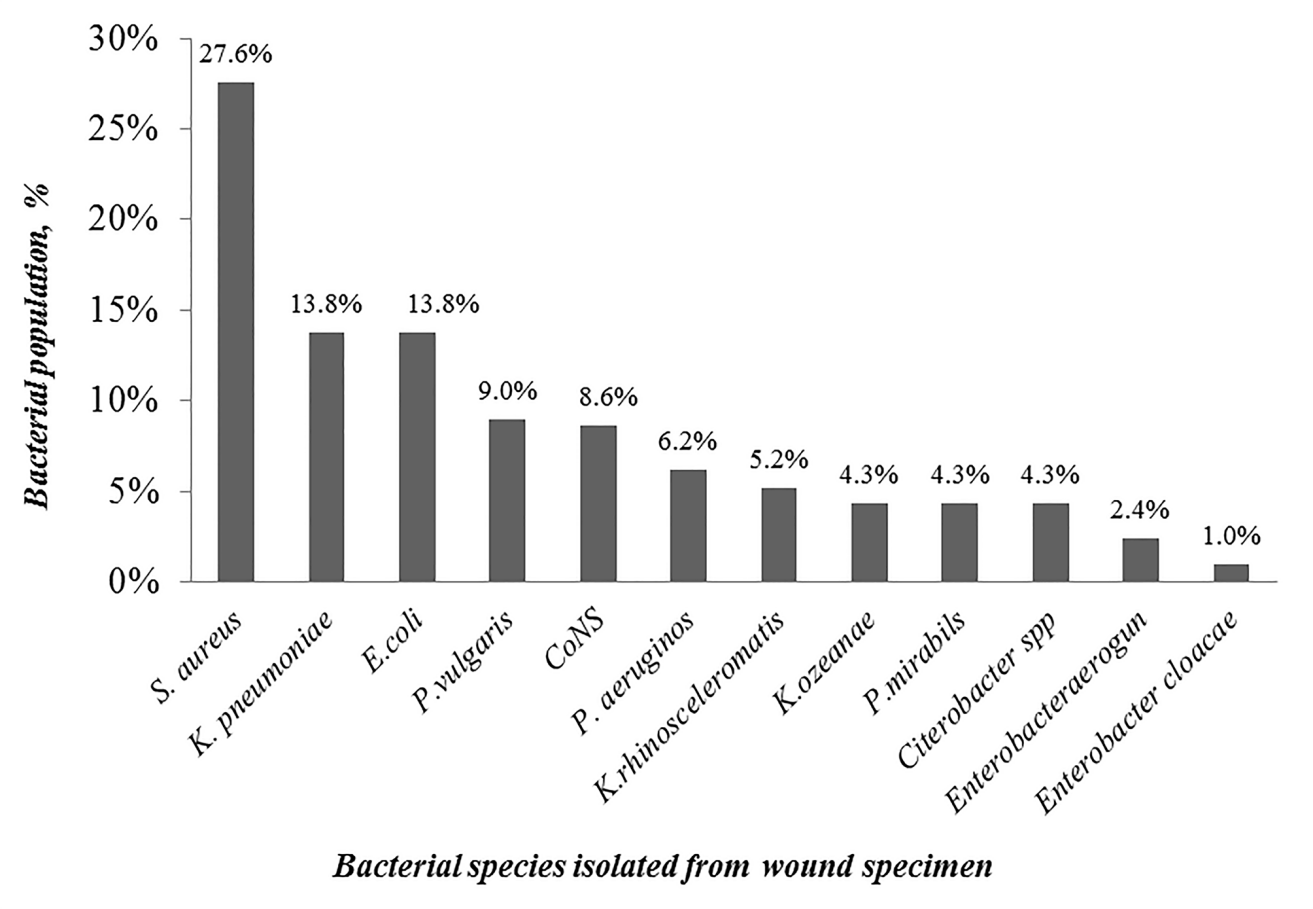
Bacterial profile of bullet-related wound infections of study subjects at selected three hospitals, in Bahir Dar Northwest Ethiopia. The bacterial species are as follows: *Staphylococcus aureus, Klebsiella pneumonia*, *Escherichia coli, Proteus vulgaris Klebsiella rhinosceleromatis, Proteus mirabils, Citerobacter spp, Enterobacter aerogenes, Enterobacter cloacae, Coagulase negative Staphylococci (CoNS), Klebsiella ozeanae* and *Pseudomonas aeruginosa*

Of the 384 swab specimens, 210 (54 0.7%) were culture positive for bacterial pathogens and 174 (45.3%) were bacteriologically sterile. The presence of only one species isolated from each sample was the most frequent (207 (98.6%)) while more than one species were isolated from only 3 (1.4%) swab specimens. From the total of 210 bacterial isolates, 134 (63.8%) were Gram-negative while 76 (32.2%) were Gram-positive. The most common Gram-negative organism cultured from bullet-related wounded patient specimens was *Klebsiella* species. It was the predominant organism isolated (49 (23.3%)) followed by *Escherichia coli* (28 (13.3%)), *Proteus* species (28 (13.3%)), *Pseudomonas* species (13 (6.2%)), Citrobacter species (9 (4.3%)) and *Enterobacte*r species (7 (3.4%)). Among the Gram-positive cocci cultured from the specimens of bullet-injured patients, the most frequently isolated organisms were *Staphylococcus aureus* (58 (27.6%)) and coagulase-negative staphylococci (CoNS) (18 (8.6%)) (Fig. 1).

A total of eleven antibiotics were used to test antimicrobial susceptibility profile of those isolated bacteria. The antibiotics were Gentamycin, Ampicillin, Oxacillin, Cefepime, Ceftazidime, Ceftriaxone, Tetracycline, Norfloxacin, Vancomycin, Erythromycin, Ciprofloxacin. The most common antibiotic-resistant Gram-negative bacteria were *Escherichia coli*, *Citerobacter* species and *Proteus* species.

*Escherichia coli* isolates were resistance to Ampicillin (100%), Cefepime (100%) Ceftazidime (100%), Ceftriaxone (100%), Tetracycline (86%), Norfloxacin (85.7%), Erythromycin (92.1%), Gentamycin (50%). The next antibiotic-resistant isolate was *Citrobacter* species which was sensitive to only Ciprofloxacin (77.8%) but resistant to other antimicrobial agents including Gentamycin (100%), Ampicillin (100), Cefepime (100%), Ceftazidime (100%), Erythromycin (100%), Ceftriaxone (89.9%) and Tetracycline (77.8%). *Proteus* species was also resistant to several antibiotics such as *P. mirabilis* (100%) to Ampicillin, *P. vulgaris* (89.5%) to Ampicillin and (100%) to Cefepime, *P. mirabilis* (100%) and *P. vulgaris* (90%) to Ceftazidime, *P mirabilis* (100%) and *P. vulgaris* (89.5%) to Ceftriaxone, *P. mirabilis* (100%) and *P. vulgaris* (94.7%) to Tetracycline, *P. mirabilis* (100%) and *P. vulgaris* (89.5%) to Erythromycin and *P. mirabilis* (66.7%) and *P. vulgaris* (64.4%) to Ciprofloxacillin. Unexpectedly, in this study, *Klebsiella* and *Pseudomonas* species were found to be sensitive to some antibiotics. For instance, about 82% *K. rhinosceleromatis, K. pneumoniae* (75.9%) and *K. ozeanae* (55.6%) isolates were sensitive to Gentamycin, *K. rhinosceleromatis (77.7%) and K. ozenae* (56%) were sensitive to Erythromycin, and *K. rhinosceleromatis* (72%), *K. pneumoniae* (62.1%) were sensitive to Ciprofloxacin. *Pseudomonas* species isolates were generally sensitive to Gentamycin (92.3%), Erythromycin (76.9%) and Ciprofloxacin (84.6%).

Among Gram positive isolates, *Staphylococcus aureus* was resistant to various antibiotics including Oxacillin (100%), Vancomycin (89.5%), Norfloxacin (87.9%), Erythromycin (87.7%), Tetracycline (65.5%), Ciprofloxacin (79.3%), Cefepime (100%), Ceftriaxone (100%) and Ceftazidime (100%) while sensitive to Gentamycin. Coagulase Negative Staphylococci (CoNS) isolates were resistant to most antibiotics employed in the study (Table 4). In this study, bacterial isolates with intermediate antibiotic resistance to antimicrobial agents were not observed.

**Table 4:** Antimicrobial susceptibility profile of organisms isolated from bullet-related bacterial wound infections in selected hospitals, Bahir Dar city, Northwest Ethiopia (*n* = 210 isolates)

AST discs	AST patterns	K. rhinosceleromatis *N* (%) (*n* = 11)	*P*. mirabils *N* (%) (*n* = 9)	Citerobacter spp *N* (%) (*n* = 9)	*P*. vulgaris *N* (%) (*n* = 19)	E. coli *N* (%) (*n* = 28)	E. aerogenes *N* (%) (*n* = 5)	E. cloacae *N* (%) (*n* = 2)	S. aureus *N* (%) (*n* = 58)	CoNS *N* (%) (*n* = 18)	K. ozaenae *N* (%) (*n* = 9)	*P*. aeruginosa *N* (%) (*n* = 13)	K. pneumoniae *N* (%) (*n* = 29)
CN	**S**	*9(81,8)*	*3(33.3)*	*0(0.0)*	*4(21.1)*	*14(50)*	*2(40)*	*1(50)*	*34(56.6)*	*0(0.0)*	*5(56.6)*	*12(91.3)*	*22(75.9)*
	**R**	*2(18.2)*	*6(66.7)*	*9(100)*	*15(78.9)*	*14(50)*	*3(60)*	*1(50)*	*24(41.4)*	*18(100)*	*4(44.4)*	*1(7.7)*	*7(24.1)*
AMP	**S**	*1(9.1)*	*0(0.0)*	*0(0.0)*	*2(10.5)*	*0(0.0)*	*2(40)*	*1(50)*	*1(1.7)*	*0(0.0)*	*0(0.0)*		*1(3.4)*
	**R**	*10(90.9)*	*9(100)*	*9(100)*	*17(89.5)*	*28(100)*	*3(60)*	*1(50)*	*57(98.3)*	*18(100)*	*9(100)*		*28(96.6)*
OXC	**S**								*0(0.0)*	*0(0.0)*			
	**R**								*58(100)*	*18(100)*			
FEP	**S**	*0(0.0)*	*0(0.0)*	*0).(0.0)*	*0(0.0)*	*0(0.)*	*0(0.00*	*0(0.0)*	*0(0.0)*	*1(5.6)*	*0(0.0.)*	*1(7.7)*	*0(0.0)*
	**R**	*11(100)*	*9(100)*	*9(100)*	*19(100)*	*28(100)*	*5(0.0)*	*2(100)*	*58(100)*	*17(93.4)*	*9(100)*	*12(91.3)*	*29(100)*
CRO	**S**	*0(0.0)*	*0(0.0)*	*1 (10.1)*	*2(10.5)*	*0(0.0)*	*0(0.0)*	*0(0.0)*	*0(0.0)*	*1(5.6)*	*0(0.0.)*	*0(0.0.)*	*5 (17.2)*
	**R**	*11(100)*	*9(100)*	*8(88.9)*	*17(88.5)*	*28(100)*	*5(100)*	*2(100)*	*58(100)*	*17(94.4)*	*9(100)*	*13(100)*	*24(82.8)*
CAZ	**S**	*0(0.0)*	*0(0.0)*	*0(0.0)*	*2 (10.5)*	*0(0.0)*	*0(0.0)*	*0(0.0)*	*0(0.0)*	*0(0.0)*	*0(0.0)*	*0(0.0)*	*1(3.4)*
	**R**	*11(100)*	*9(100)*	*9(100)*	*17(89.5)*	*28(100)*	*59,100)*	*2(100)*	*58(100)*	*18(100)*	*9(100)*	*13(100)*	*28(95.6)*
TE	**S**	*8(72.7)*	*0(0.0)*	*2 (22,2)*	*1(5.3)*	*4 (13.3)*	*0(0.0)*	*0 (0.0.)*	*20(35.5)*	*9(50)*	*1(11.1)*	*6(53.8)*	*9 (31)*
	**R**	*3(27.3)*	*9(100)*	*7(76.8)*	*18(94.7)*	*24(85.7)*	*5(100)*	*2(100)*	*38(65.5)*	*9(50)*	*8 (88.9)*	*7(46.2)*	*20 (69)*
Nor	**S**	*5(45.5)*	*0(0.0)*	*1(11.1)*	*0(0.0)*	*4(14.3)*	*1 (20)*	*1(50)*	*7(12.1)*	*2(11.1)*	*0(0.0)*	*8(61.5)*	*7(24.1)*
	**R**	*6(55.)*	*9(100)*	*8(88.9)*	*19(100)*	*24(85.7)*	*4(80)*	*1(50)*	*51(87.9)*	*16(88.9)*	*9(100)*	*5(38.5)*	*22(75.9)*
Van	**S**								*6(10.5)*	*1(5.6)*			
	**R**								*52(89.5)*	*17(93.4)*			
E	**S**	*8(72.7)*	*0(0.0)*	*0(0.0)*	*0(0.0)*	*5(17.9)*	*2(40)*	*1(50)*	*7(12.3)*	*0(0)*	*5(55.6)*	*5(55.6)*	*12(41.4)*
	**R**	*3(27.3)*	*9(100)*	*9(100)*	*9(100)*	*23(82.1)*	*5(60)*	*1(50)*	*51(87.7)*	*18(100)*	*4(44.4)*	*4(44.4)*	*17(48.6)*
CIP	**S**	*8(72.7)*	*3(33.3)*	*7(77.8)*	*6(31.3)*	*13(46.4)*	*3(60)*	*2(100)*	*12(20.7)*	*2(11.1)*	*3(33.3)*	*11(84.6)*	*18(62.1)*
	**R**	*3(27.3)*	*6(64.7)*	*2 22.2)*	*13(68.7)*	*15(53.6)*	*2(40)*	*0(0.0)*	*46(79.3)*	*16(89.9)*	*6(66.7)*	*2(15.4)*	*11(37.9)*

**Key: S** = sensitive, **R** = resistant, **CN** = Gentaycin, **AMP** = Ampicillin, **OXC** = Oxacillin, **FEP** = Cefepime, **CAZ** = Ceftadizime, **CRO** = Ceftriaxone, **TE** = Tetracycline, **Nor** = Norfoxacin, **Van** = vancomycin, **E** = erythromycin, **CIP** = Ciprofloxacin

### Multidrug resistance profile of bacterial isolates

In the current study, isolates which didn’t respond to three or more antibiotics were considered as multidrug-resistant [22]. Hence, among Gram-negative bacterial isolates, *K. rhinoscleromatis* (7/11(63.6%)), *K. ozeanae* (9/9(100)), *K. pneumoniae* (25/29(86.2%)), *P. aeruginosa* (5/13(38.5%)), *P. mirabilis* (7/9(87.5%)), *Citrobacter* species (9/9(100)), *P. vulgaris* (18/19(94.7%)), *E. coli* (27/28(96.4%)), *Enterobacter aerogens* (4/5(80.0%)), *Enterobacter cloacae* (1/2(50%)) were categorized in to multi-drug resistant isolates. From the Gram-positive isolates, *Staphylococcus aureus* (57/58(98.3%)), CoNS (17/18(94.4%)). As a result, the overall multi-drug resistant rate was 97.6%. The most frequently isolated Gram-negative MDR bacteria were *Citrobacter* species, *E. coli, Proteus* species and *Enterobacter* species. Among the Gram-positive cocci, *Staphylococcus aureus* and Coagulase negative Staphylococcus isolates were resistant to multiple antibiotics employed in this study (Table 5).

**Table 5:** Multidrug resistance profile of bacterial isolates with ≥ 3 antimicrobial agents isolated from bullet-related wound infection

Isolated bacteria	No. of isolates	MDR isolates *N*(%)			
		R3	R4	R5	R6
*K. rhinosceleromatis*	11	-	-	4(36.4)	7(63.6)
*P. mirabils*	8	-	-	1(12.5)	7(87.5)
*Citrobacter species*	9	-	-	1(11.1)	8(88.9)
*P. vulgaris*	19	-	-	2(10.5)	17(89.5)
*E. coli*	28	-	-	1(3.6)	27(96.4)
*Enterobacter aerogenes*	5	-	-	1(20)	4(80)
*Enterobacter cloacae*	2	-	-	1(50)	1(50)
*S. aureus*	58	-	-	1(1.7)	57(98.3)
*CoNS*	18	-	1(5.6)	-	17(94.4)
*K. ozaenae*	9	-	-	-	9(100)
*P. aeruginosa*	13	-	5(38.5)	3(23.1)	5(38.5)
*K. pneumoniae*	25	1(3.6)	2(7.1)	2(7.1)	23(82.1)

### Factors associated with bullet-related bacterial wound infections

Based on the univariate analysis of bullet-related bacterial isolates, variables such as contamination of bullet wounded site, location of the wound, hospitalization, types of the bullet and khat chewing were selected for possible inclusion in multivariate logistic regression models to avoid confounding effects. The multivariate analysis specified that bullet-related bacterial isolates were significantly associated with habit of cigarette smoking (AOR = 1.43, 95%CI(0.195,1.946), p-value = 0.036), soil contamination of bullet-wounded site (AOR = 4.55, 95%CI(1.086,19.098), p-value = 0.038) and hospitalization stay (AOR = 3.147, 95%IC(0.436,6.896), p-value = 0.004). Patients who had soil contamination upon bullet injury were about 5 times more likely to have bacterial wound infections compared to other contaminants. Patients’ hospitalization stay for more than 2 months was found 3 times more likely to cause bacterial wound infections than patients stayed in hospital for fewer than 2 months (Table 6).

**Table 6:** Factors associated with bullet-related wound bacterial infection in selected hospitals of Bahir-Dar city, Northwest Ethiopia, 2022 (*n* = 384)

Characteristics		No.	Culture positive *N*(%)	COR (95%CI)	*p*-value	AOR (95%CI)	*p*- value
Cigarette Smoking	Yes	65	43(66.2)	1.779(1.017, 3.111)	0.043	1.430(0.195, 1.946)	0.036*
	No	319	167(79.5)	1			
Khat Chewing	Yes	48	30 (14.3)	0.692(0.272, 1.290)	0.247	0.562(0.213, 1.124)	0.065
	No	336	18 (10.3)	1			
Contamination of wounded site	Soil	251	128(510	2.522(1.077, 5.908)	0.033	4.554(1.086, 19.09)	0.038*
	Water	24	15(62.5)	1.175(0.494, 5.025)	0.443	2.11(0.964, 2.346)	0.56
	Dust	62	41(66.1)	1.345(0.510, 0.354)	0.004	0.232(1.012, 3.456)	0.54
	Mud	18	5(27.8)	1			
Hospitalization Stay	7 days	73	37(50.7)	1			
	15 days	80	50(62.5)	0.981(0.844, 1.140)	0.800	1.230(0.134, 2.134)	0.09
	30 days	76	42(56.0)	1.817 (0.864, 3819)	0115	1.021(0.209, 0.832)	0.074
	60 days	103	44(42.7)	3.101(1.533, 6.273)	0.002	3.147(1.436, 6.896)	0.004**
	90–180 days	53	37(69.80)	3.101(1.533, 6.273)	0.05	2.453(0.675, 2.123)	0.09
Types of bullet	Blast	35	20(9.5)	1.591(0.095, 26.761)	0.747	0.534(0.5012, 1,322)	0.85
	Gunshot (pistil)	50	26(12.5)	1.193(0.507, 2.909)	0.666	1.256(1.432, 3.457)	0.07
	Artillery bullet	6	6(2.9)	1.468(0.680, 3.170)	0.328	0.980(0.324, 0.590)	0.09
	Sniper bullet	51	34(16,2)	0.0(0.00)	0.999	0.870(0.265, 0.732)	0.08
	SKS –bullet	9	2(1.0)	0.795(0.361, 1.752)	0.570	0.690(1.431, 4.098)	0.07
	Kalashnikov bullet	159	80(38.1)	5.566(1.059, 29.270)	0.43	0.875(0.236, 0.704)	0.08
	Mortar bullet	15	5(2.9)	1.571(0.847, 2.912)	0.12	0.745(0.323, 0.820)	0.45
	Rocket bullet	2		1			

* *p-value* < 0.05, ** *p-value* < 0.01

## Discussion

The prevalence of bullet-related bacterial wound infections in three selected hospitals in Bahir Dar city was 54.7% where bacterial wound infection is still one of the main causes of hospitalizations in the study area with high possibilities of morbidity and mortality even with the current advancement of antimicrobial agents. From the total of 210 culture-positive specimens, 134 (63.8%) were Gram-negative bacteria and 76 (36.2%) were Gram-positive. This indicated that the most frequently isolated bacteria from bullet-related wounds were Gram-negative bacteria including *Klebsiella* species (49(23.3%)), *Escherichia coli* (28(13.3%)), *Proteus* species (28(13.3%)), *Pseudomonas* species (13(6.2%)), *Citerobacte*r species (9(4.3%)) and *Enterobacte*r species (7(3.4%)) while isolates of Gram-positive cocci were *Staphylococcus aureus* (58(27.6%)) and coagulase negative *Staphylococci* (18(8.6%)). These findings were also reported by previously conducted studies in Ukraine [2, 5].

Majority of swab specimens (207(98.6%)) of bullet-injured wounds exhibited only one bacterial species while more than one bacterial species were isolated from few number of wound specimens (3(1.4%)). This data was also demonstrated by other studies done in Ukraine [5]. In the current study, among Gram-positive isolates, *Staphylococcus aureus* (58(27.6%)) and coagulase-negative *Staphylococci* species (18(8.6%)) were frequently isolated organisms from wound specimens. This was also explained by other studies conducted previously in the USA [11, 23]. Antimicrobial-resistant bacteria caused illnesses can produce more severe symptoms than their predecessors. Even though novel antibiotics have shown considerable promise against antibiotic-resistant bacteria, rapid diagnosis remains a serious challenge due to antimicrobial resistance bacteria [24]. In this study, Gram negative bacterial isolates such as *Escherichia coli* (96.4%), *Proteus* Species (92.6%), *Citrobacter* Species (100%), *Enterobacter* species (71,4%), *K. pneumoniae* (86.2%) and *P. aeruginosa* (38.5%) were resistant to most antibiotics (Gentamycin, Ampicillin, Cefepime, Ceftriaxone, Ceftazidime, Tetracycline, Norfloxacin, Erythromycin, and Ciprofloxacin) while the Gram positive cocci, *Staphylococcus aureus* (98.3%), were resistance to Oxacillin, Ampicillin, Cefepime and Ceftriaxone. The previous studies conducted in Syria also showed that the most common Gram negative isolated bacteria were *E. coli* (100%), *Proteus* species (63%), *Enterobacter* species (78%), *Klebsiella pneumoniae* (82%), *Pseudomonas* species (17%) and the Gram positive cocci, *Staphylococcus aureus* (73%), was resistant to the most antimicrobials [17, 19] which is a relatively different compared to this study. This variation might be due to the difference in the type of injuries, site of injuries, awareness to wound contamination and geographic locations.

Among the bacterial isolates of bullet-related wound infections, *Pseudomonas* and *Klebsiella* species isolates were sensitive to most frequently used antibiotics in the study area. These species were sensitive to Gentamycin, Erythromycin and Ciprofloxacin. The Gram positive cocci including *Staphylococcus aureus* and coagulase negative *Staphylococci* isolates were resistant to Oxacillin and Vancomycin and also resistant to most broad-spectrum antibiotics tested in the study. This is explained by a previous study conducted in Iraq [11, 17]. However, bullet-related bacterial wound infections have not yet adequately characterized the trends of infectious complications and associated outcomes in injured personnel during the conflict situations [25].

Multidrug resistance profiles of bacterial isolates to most antibiotics tested were observed in the present study. This was in agreement with the previous study conducted in two countries Iraq and Afganistan [17]. Gram negative bacterial isolates resistant to more than or equal to three antibiotics were *K.rhinosceratis* (7/11(63.6%)), *K.ozeanae* (9/9(100%)), *K. pneumoniae* (25/29(86.2%)), *P. aeruginosa* (5/13(38.5%)), *P. mirabilis* (7/9(87.5%)), *Citerobacter* species (9/9(100%)), *Proteus vulgaris* (18/19(94.7%)), *E.coli* (27/28(96.4%)), *Enterobacter aerogens* (4/5(80.0%)), *Enterobacter cloacae* (1/2(50%)) while the Gram positive cocci were *Staphylococcus aureus* (57/58(98.3%)) and CoNS (17/18(94.4%)).

Factors associated with the development of bullet-related bacterial wound infections include wound type and severity, site and location of the wound, individuals’ habit, the presence of embedded foreign material or fragments such as injured personnel’s clothing, dirt, and debris, initiation of antimicrobial agents, prior antimicrobial pressure, hospitalization and the presence of nosocomial pathogens, especially multidrug-resistant pathogens at treatment facilities [1, 26]. As indicated in the previous study done in Beirut [27], we found that hospitalization of bullet-injured personnel was significantly associated with bacterial isolates of bullet-related wounds. Contamination of bullet-injured wounds with environmental materials including personnel closes, dust or soil was also associated with the bacterial isolates of wound infections which is in agreement with other study conducted in the USA [11]. The other factor associated with bacterial isolates was the smoking habit of bullet-injured personnel though most of study participants in our study never smoked cigarates.

### Limitations of the study

Unavailaibility of documented history of prophylactic use of antibiotics and period of stay at the site of combat after bullet injury and anaerobic bacterial pathogens that could be difficult to culture in our laboratory setting were among the limitations of the current study.

## Conclusions

The prevalence of bacterial isolates among bullet-related wound patients was 54.7%. The most frequently isolated bacteria was *S. aureus* followed by *Klebsiella* species, *E. coli, Proteus* species and CoNS. These isolates were MDR to ≥ 3 antibiotics employed in the study. Hence, avoiding soil or other contaminants upon bullet-injury, prompt investigation of bullet-related bacterial wound infections, monitoring of their drug sensitivity patterns, proper antibiotic usage and infection prevention practice awareness creation in vulnerable personnels are essential to reduce mortality and morbidity associated with bullet-related wound bacterial infection.

## Data Availability

The study materials and data are available from the corresponding author upon request.

## Abbreviations

AMR: Anti-Microbial Resistance
AST: Antimicrobial Susceptibility Test
CLSI: Clinical Laboratory Standard Institute
CoNS: Coagulase Negative Staphylococci
FHCSH: Felege Hiwot Comprehensive Specialized Hospital
MDR: Multidrug Resistance
NWCL3BH: North West Command Level3 Bahir Dar Hospital
APHI: Amhara Public Health Institute
PTO: Post-Traumatic Osteomyelitis
QC: Quality Control
